# Case Report: A Case Report of Stereotactic Ventricular Arrhythmia Radioablation (STAR) on Large Cardiac Target Volume by Highly Personalized Inter- and Intra-fractional Image Guidance

**DOI:** 10.3389/fcvm.2020.565471

**Published:** 2020-11-23

**Authors:** Maria Lucia Narducci, Francesco Cellini, Lorenzo Placidi, Luca Boldrini, Francesco Perna, Gianluigi Bencardino, Gaetano Pinnacchio, Roberta Bertolini, Giorgio Cannelli, Vincenzo Frascino, Luca Tagliaferri, Silvia Chiesa, Gian Carlo Mattiucci, Mario Balducci, Maria Antonietta Gambacorta, Marco Rossi, Luca Indovina, Gemma Pelargonio, Vincenzo Valentini, Filippo Crea

**Affiliations:** ^1^Dipartimento di Scienze Cardiovascolari, Fondazione Policlinico Universitario A. Gemelli IRCCS, Rome, Italy; ^2^Dipartimento di Diagnostica per Immagini, Radioterapia Oncologica ed Ematologia, UOC di Radioterapia Oncologica, Fondazione Policlinico Universitario A. Gemelli IRCCS, Rome, Italy; ^3^Istituto di Radiologia, Università Cattolica del Sacro Cuore, Rome, Italy; ^4^Department of Anesthesiology, Intensive Care and Pain Therapy, Fondazione Policlinico A. Gemelli IRCCS, Catholic University of the Sacred Heart, Rome, Italy; ^5^Istituto di Cardiologia, Università Cattolica del Sacro Cuore, Rome, Italy

**Keywords:** ventricular arrhythmia, SBRT, cardiac radioablation, radiosurgery, ventricular tachycardia, radiotherapy, personalized medicine, STAR

## Abstract

**Introduction:** Although catheter ablation is the current gold standard treatment for refractory ventricular arrhythmias, sometimes its efficacy is not optimal and it's associated with high risks of procedural complication and death. Stereotactic arrhythmia radioablation (STAR) is increasingly being adopted for such clinical presentation, considering its efficacy and safety.

**Case Presentation:** We do report our experience managing a case of high volume of left ventricle for refractory ventricular tachycardia in advanced heart failure patient, by delivering a single fraction of STAR through an highly personalization of dose delivery applying repeated inter- and continuous intra-fraction image guidance.

**Conclusion:** According to the literature reports, we recommend considering increasing as much as possible the personalization features and safety technical procedure as long as that is not significantly affecting the STAR duration. Moreover, the duration in itself shouldn't be the main parameter, but balanced into the frame of possibly obtainable outcome improvement. At best of our knowledge, this is the first report applying such specific technology onto this clinical setting. Future studies will clarify these issues.

## Introduction

**Figure 1:**
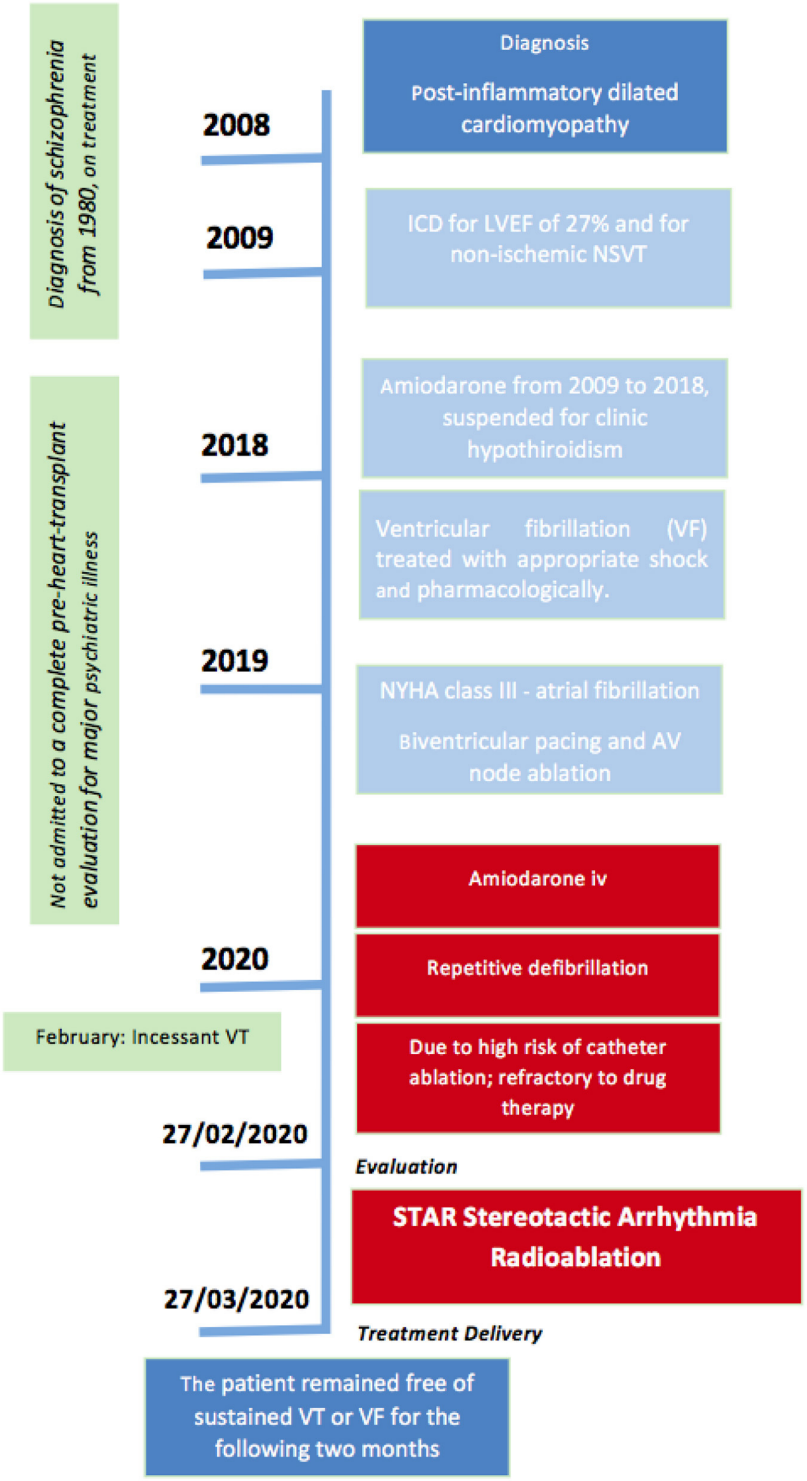
Timeline (according to CARE indications).

This case report was prepared following the CARE Guidelines (1). Patient provided informed consent to treatment and research applications. Figure 1 summarizes the Timeline of the reported clinical case. Written, informed consent was obtained from the participant for the publication of this case report (including all data and images).

### Clinical and Scientific Background

Stereotactic body radiation therapy (SBRT) is increasingly being adopted in non-oncologial framework for the non-invasive management of refractory ventricular tachycardia (VT) (2). Catheter ablation is the current gold standard treatment for VT in several cardiomyopathies (3), nevertheless its efficacy is limited by anatomical findings of reentry and not rarely associated with high risks of procedural complication and death (4, 5). Recent case series (CS) report about the efficacy and safety of stereotactic arrhythmia radioablation (STAR) (6, 7), but these reports collect together <30 patients. To this regard, some case reports (CR) also provided insights in this regard addressing different perspectives of this new approach (i.e., STAR) for a well-established procedure (i.e., SBRT) (8, 9). Still many issues about details of STAR have been not standardized, including: the ideal workflow, the radiation therapy best performing machine, definition of margins expansion for target delineation, the indications about the best image guidance limiting procedural error. Thus, providing insights by case per case clinical experience can contribute to the addressing the standard definition for STAR. We do report our experience managing a case of high volume of left ventricle for refractory ventricular tachycardia in advanced heart failure patient, by delivering a single fraction of STAR through an highly personalization of dose delivery applying repeated inter- and continuous intra-fraction image guidance. Due to the peculiarity of the clinical presentations we meant to apply the most dedicated Linear Accelerator (Linac)-based technology at our disposal for both stereotactic high-conformal dose delivery and image guidance applied to intra- and inter-fraction monitoring. We performed SBRT by TrueBeam Edge Linac (Varian Medical Systems, Palo Alto, CA). At best of our knowledge, this is the first report applying such specific technology onto this clinical setting.

### Case Presentation—Patient History

A 60-year-old man, suffering from a post-inflammatory dilated cardiomyopathy since 2008, was admitted on February 2020 to the ICU because of an electrical storm (ES) due to incessant VT rapidly degenerating to ventricular fibrillation (VF) and consequent appropriate shock.

The patient presented with diagnosis of schizophrenia from 1980 on actual treatment with lusiradone 18.5 mg/day, sertralin 50 mg/day, lorazepam 2 mg/day.

He was admitted on December 2008 for dyspnea after previous flu-like syndrome. At baseline echocardiogram severe left ventricular dilation and global hypokinesia were found, with left ventricular ejection fraction (LVEF) of 27%. A cardiac magnetic resonance imaging (cMRI), performed in 2008, showed left ventricular dilation with global hypokinesia and late gadolinium enhancement of basal posterior wall suggestive of a post-inflammatory cardiomyopathy. A coronary angiography was performed without evidence of coronary arteries stenosis. Consequently, he had been implanted with an implantable cardioverter-defibrillator (ICD) for primary prevention. He was on effective antiarrhythmic therapy with amiodarone from 2009 to 2018. Amiodarone was suspended for clinic hypothiroidism. On 2019 the patient was admitted to our Department for acute decompensated heart failure (NYHA class III) due to atrial fibrillation (Afib) and high rate response; coronary angiography was normal. During this hospitalization, a pre-heart-transplant evaluation has been required: for major psychiatric illness that cannot be managed sufficiently to allow post-transplant care and safety, the patient was not admitted to a complete pre-heart-transplant evaluation. Upgrade procedure of biventricular pacing and atrioventricular node ablation was performed without complications.

On February 2020 the patient presented with multiple episodes of VT LC 390 msec and VF interrupted by shock. Amiodarone (starting dose 75 mg/h intravenous -iv-) was started at admission in ICU. After amiodarone iv, the patient was stabilized whereas baseline LVEF was 12%, the left ventricular outflow tract velocity time integral (LVOT VTI) was 6.7 cm, with a stroke volume index (SVi) of 15 ml/m2 with consequent reduced systemic perfusion. Right ventricular dysfunction was indicated by tricuspid annular plane excursion (TAPSE) 13 mm, S'TDI 7 cm/sec, right ventricular fraction area change (RVFAC) 12%.

**Figure 2:**
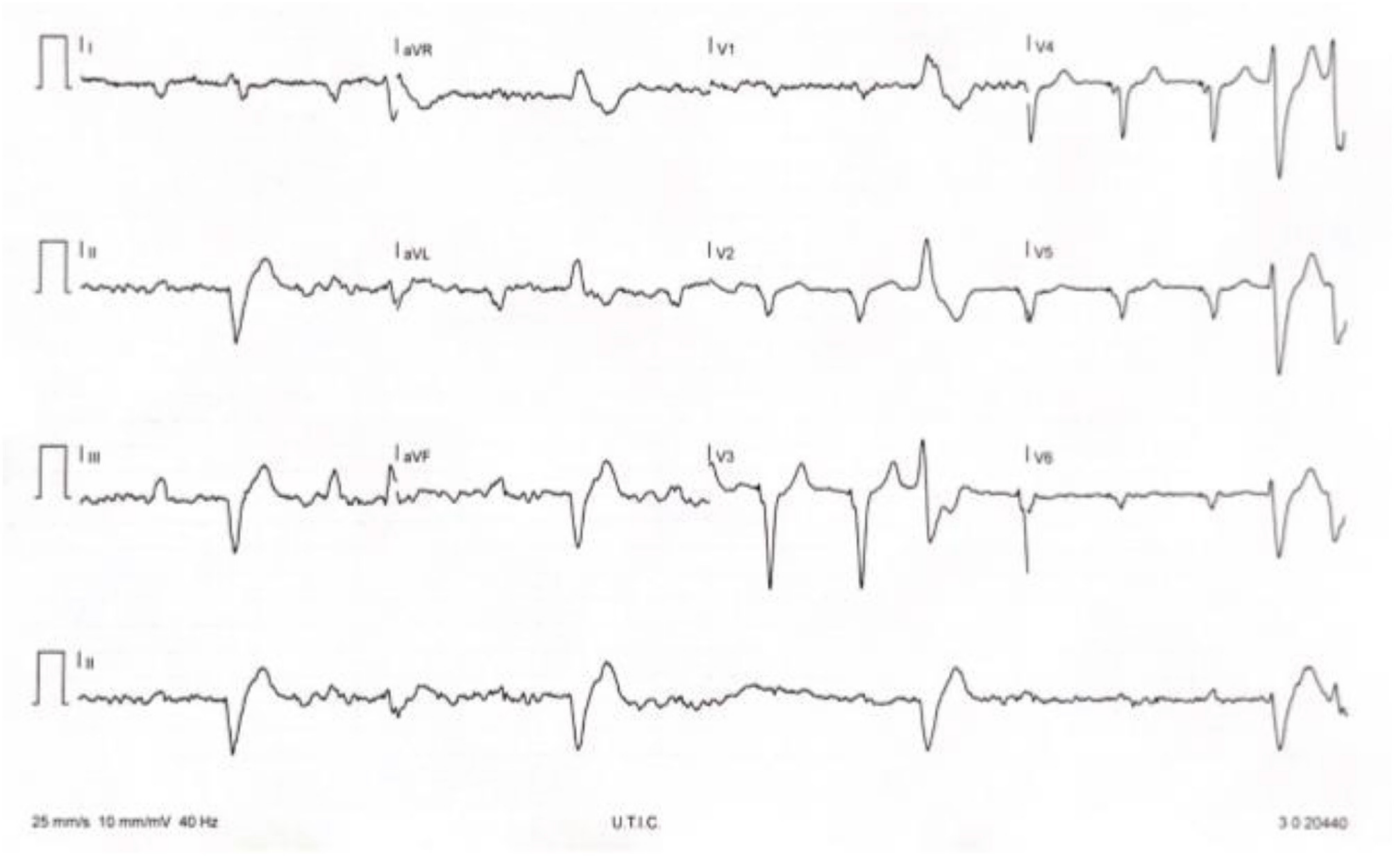
Patient monomorphic PVC.

Moderate mitralic regurgitation and mild tricuspid regurgitation was detected with associated significant pulmonary hypertension. After 48 h in ICU amiodarone was interrupted for QT prolongation (QTc 497 msec) and neurological therapy was revised with interruption of lurasidone 18.5 mg/day and introduction of low dose of risperidone (1 mg/day). Considering that monomorphic PVC consistently preceded predominantVT occurrence, we decided to attempt catheter ablation (Figure 2). The high risk for an epicardial approach with hemodynamic support in a patient with advanced biventricular dysfunction was discussed in heart team and with the patient and his family. Epicardial approach during sedation and with hemodynamic support was considered at very high risk (PAINESD risk score 17) and after complete information the patient did not provide informed consent to this approach but only for endocardial approach. After endocardial ablation, not hemodinamically tolerated sustained VT remained uncontrolled and STAR was proposed to patient. After his given informed consent STAR was planned.

## Materials and Methods (Therapeutic Interventions)

### 3D Electroanatomic Mapping

**Figure 3:**
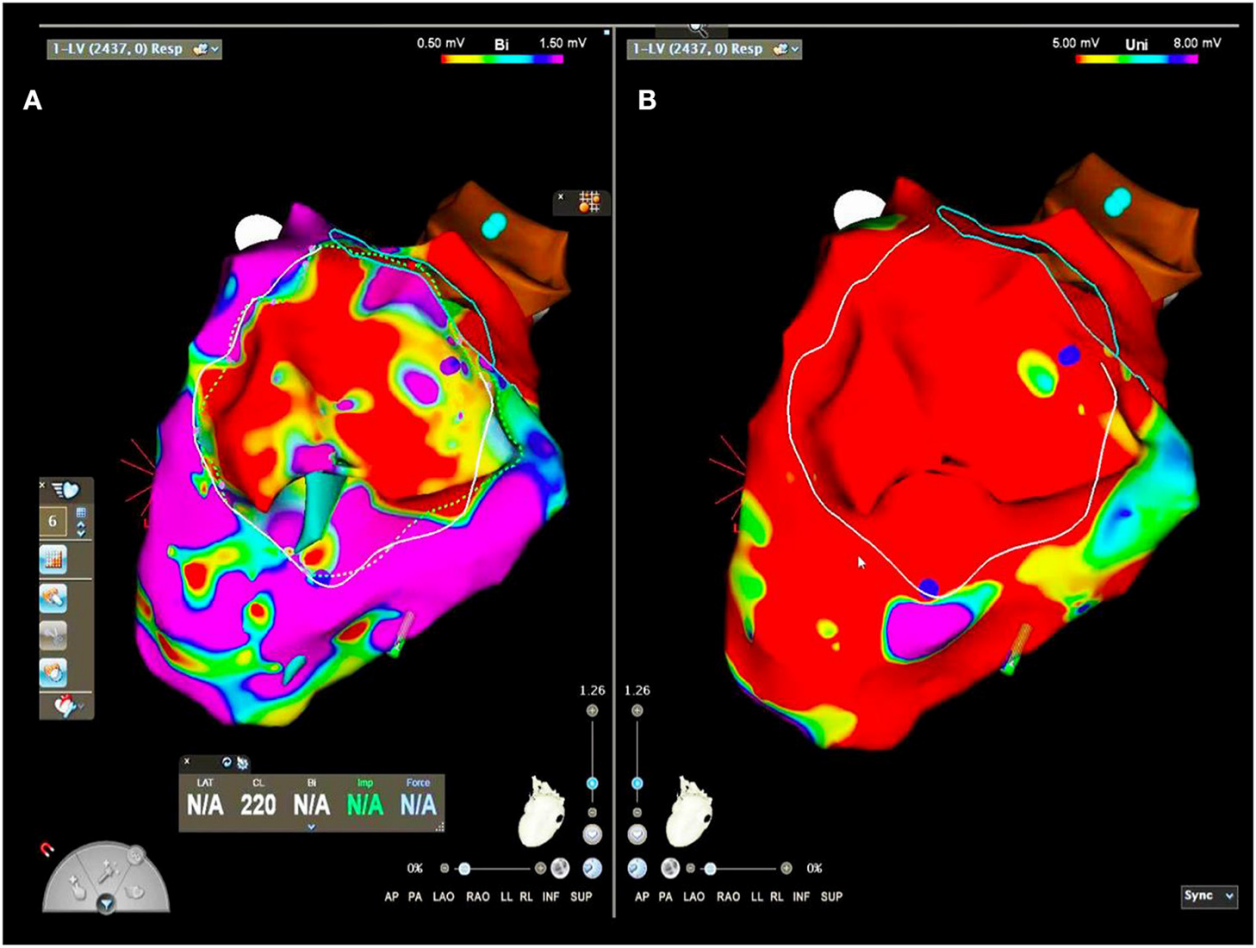
Left ventricular electroanatomic bipolar **(A)** and unipolar **(B)** mapping.

Left ventricular endocardial substrate mapping, perfomed by multipolar diagnostic catheter (Pentaray, Carto, Biosense Webster), showed basal inferior and lateral scar area (bipolar voltage <0.5 mV) (Figure 3). Intracardial echocardiography confirmed inferior scar (Video 1). Unipolar scar area (unipolar voltage <5.5 mV) was larger than bipolar scar area (respectively, 55 vs. 15 cm2) involving lateral and inferior wall from basal to apical regions. Low abnormal voltage areas or late potentials were not recorded by endocardial mapping. Consequently, catheter ablation was perfomed endocardially by 8F irrigated ablator Smarttouch, based on pacemapping correlation index. According to the patient, epicardial mapping, and ablation was not perfomed, limiting ablation success rate.

### Radiotherapy: Planning and Delivery for Stereotactic Arrhythmia Radioablation

STAR was set-up through our regular workflow including: Radiotherapy Treatment Simulation; Radiotherapy Treatment Planning; Radiotherapy Quality assurance procedures preliminary to treatment delivery; Treatment Delivery.

Three days after ablative procedure, a planning cardiac computed tomography (CT) was performed.

Magnetic Resonance Imaging (MRI) was not performed for no MRI conditional ICD. 3DEAM imaging with cardiac TC were synchronized (Figure 3).

### STAR: Treatment Simulation

The patient has been positioned in the supine position with arms raised above the head. The selected patient immobilization system employed for the simulation and the further delivery was vac-lok cushions (CIVCO Vac-LokTM Cushions). Planning CT (GE, Optima CT580 W, HiSpeed DX/I Spiral) without contrast agent has been acquired.

Multiple CT acquisition has been performed for simulation: (i) Free breathing CT; (ii) Four dimensional (4D)CT; (iii) Deep inspiration Breath hold CT (DIBH).

Based on the evaluation of the acquired CT protocol, DIBH was excluded as gating technique for the treatment delivery, since the patient was not compliant enough. Average CT, computed by the 4DCT, was instead selected for target (in term of Internal Target Volume -ITV-) and Organ at Risk (OAR) delineation, with a slice thickness of 2.5 mm.

### STAR: Target Definition and Delineation

Target delineation was defined by 3 separated rounds of delineation. The radiation oncologist (RO) first checked with cardiologist (Ca) the electroanatomic mapping. Then RO delineated the target area on the simulation CT. Finally the Target Area (TA) was simultaneously double-checked by RO and Ca for fine optimization. A margin of 3 mm was provided to TA to obtain Planning Target Volume (PTV). The final PTV volume for our STAR was 303 cc (Table 1).

**Table 1:** Dose statistic (minimum, maximum, and mean dose) of target and OARs.

**Structure**	**Volume (cc)**	**Min dose (Gy)**	**Max dose (Gy)**	**Mean dose (Gy)**
PTV	303.0	18.06	34.30	29.67
Spina canal	4.0	0.05	2.59	0.67
Heart	1816.6	0.47	34.30	11.95
Esophagus	42.8	0.06	5.01	1.52
Right lung	2466.8	0.04	10.11	1.57
Left lung	1,968	0.05	28.96	4.40
Lung	4434.8	0.04	28.96	2.83
Pericardio	339.3	0.47	34.30	8.99
Trachea	40.1	0.05	0.77	0.28
Aorta	141.6	0.08	5.05	1.39
Camera 1	172.9	0.66	13.32	5.30
Camera 2	324.5	0.65	31.23	7.60
Camera 3	235.9	1.01	19.52	10.58
Camera 4	678.6	1.27	34.30	21.11
PMK	–	0.05	0.18	0.12

### STAR: Treatment Planning

**Figure 4:**
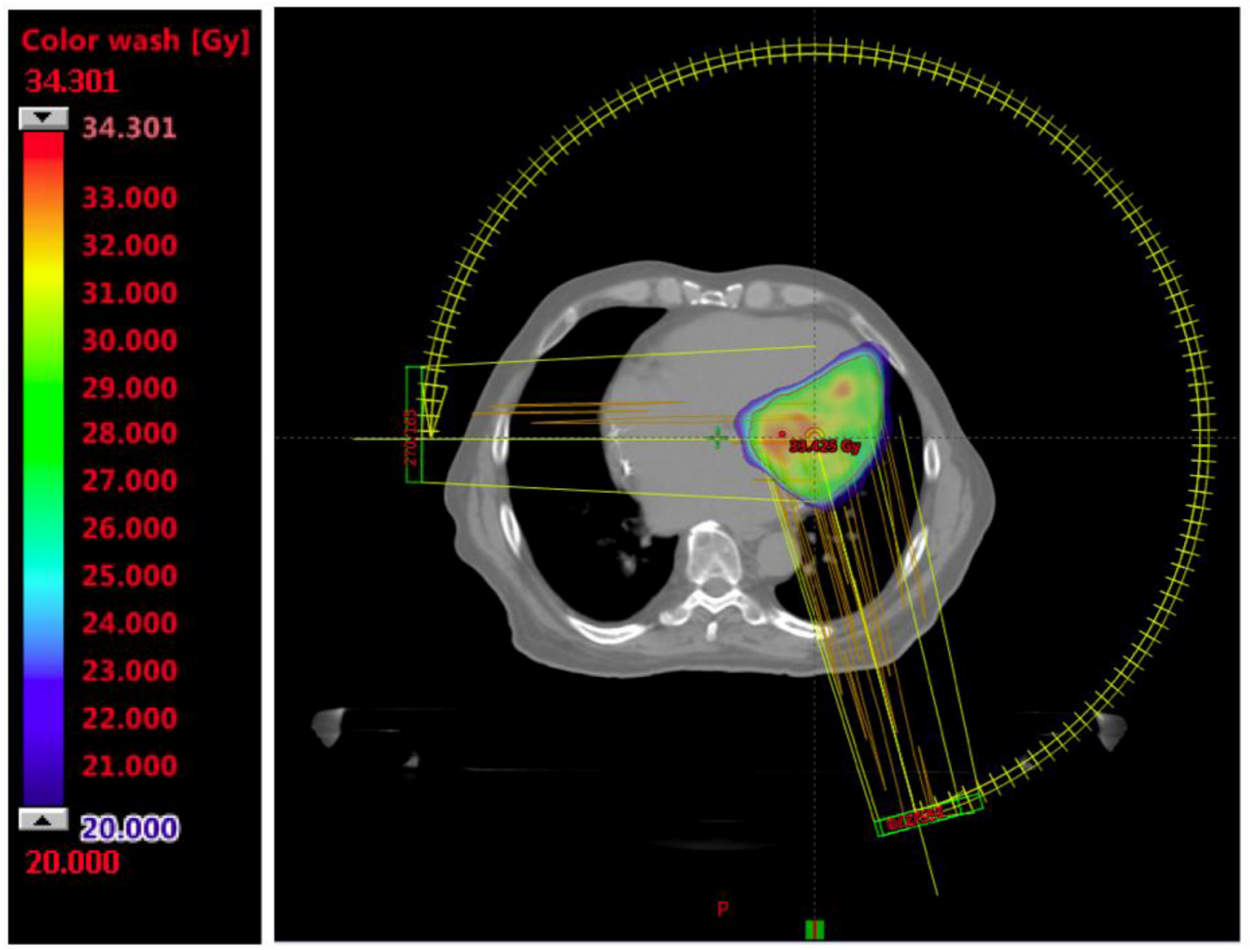
STAR Treatment Plan.

STAR was planned in one fraction using the TrueBeam Edge Linac (Varian Medical Systems, Palo Alto, CA) with 6 MV flattening filter free photons and dose calculation algorithm Acuros (Eclipse Version 15.6.04 Varian Medical Systems, Palo Alto, CA). The prescribed dose was 25 Gy to the 80% isodose, an example is shown in Figure 4.

Volumetric Modulated Arc Therapy (VMAT) was used with 3 partial arcs (PA) of 255° each to a total 11,539 monitor units: PA1 = 3,816 Monitor Units (MU) with a collimator rotation of 30°and a gantry rotation from 165 to 270° counter-clockwise (CCW), PA2 = 4,001 MU with a collimator rotation 330° and a gantry rotation from 270 to 165° clockwise (CW), PA2 = 3,722 MU with a collimator rotation of 10° and a gantry rotation from 165 to 270° CCW. All constraints were within the tolerance based on the AAPM TG101 report (10), except for the pericardia that has been optimized, decreasing the dose as low as reasonably achievable, without compromising target coverage. Pacemaker PMK maximum dose was 0.184 Gray (Gy) and the distance between PKM and PTV was 11.5 cm.

Dose statistic (minimum, maximum, and mean dose) of target and OARs is reported in Table 1.

### STAR: Quality Assurance

Patient specific pre-treatment quality assurance (QA) verification for VMAT using fluence maps measured with an electronic portal imaging device (EPID) was performed. Fluence maps for each PA was then analyzed in the Portal Dosimetry Application (Varian Medical Systems, Palo Alto, CA) with the following gamma analysis criteria: 3%, 3 mm (threshold 10% of maximum dose). Gamma passing rate, for all the three PA, always exceeded the 99%.

### STAR: Treatment Delivery

The patient was treated without the use of either sedation or anesthesia. Image guidance was performed by both volumetric imaging (by Cone Beam CT -CBCT-) before each PA delivery for positioning verification and correction, and by optical surface monitor system (OSMS) for continuous positioning intrafraction tracking and delivery triggering.

The patient was aligned at the isocenter, and 3-dimensional volumetric image guidance through CBCT was acquired. Three-dimensional alignment was performed progressively matching for alignment against the reference images of the treatment plan: bone structures, then organs (e.g., lungs), whole heart, then finally the Clinical Target Volume (CTV).

**Figure 5:**
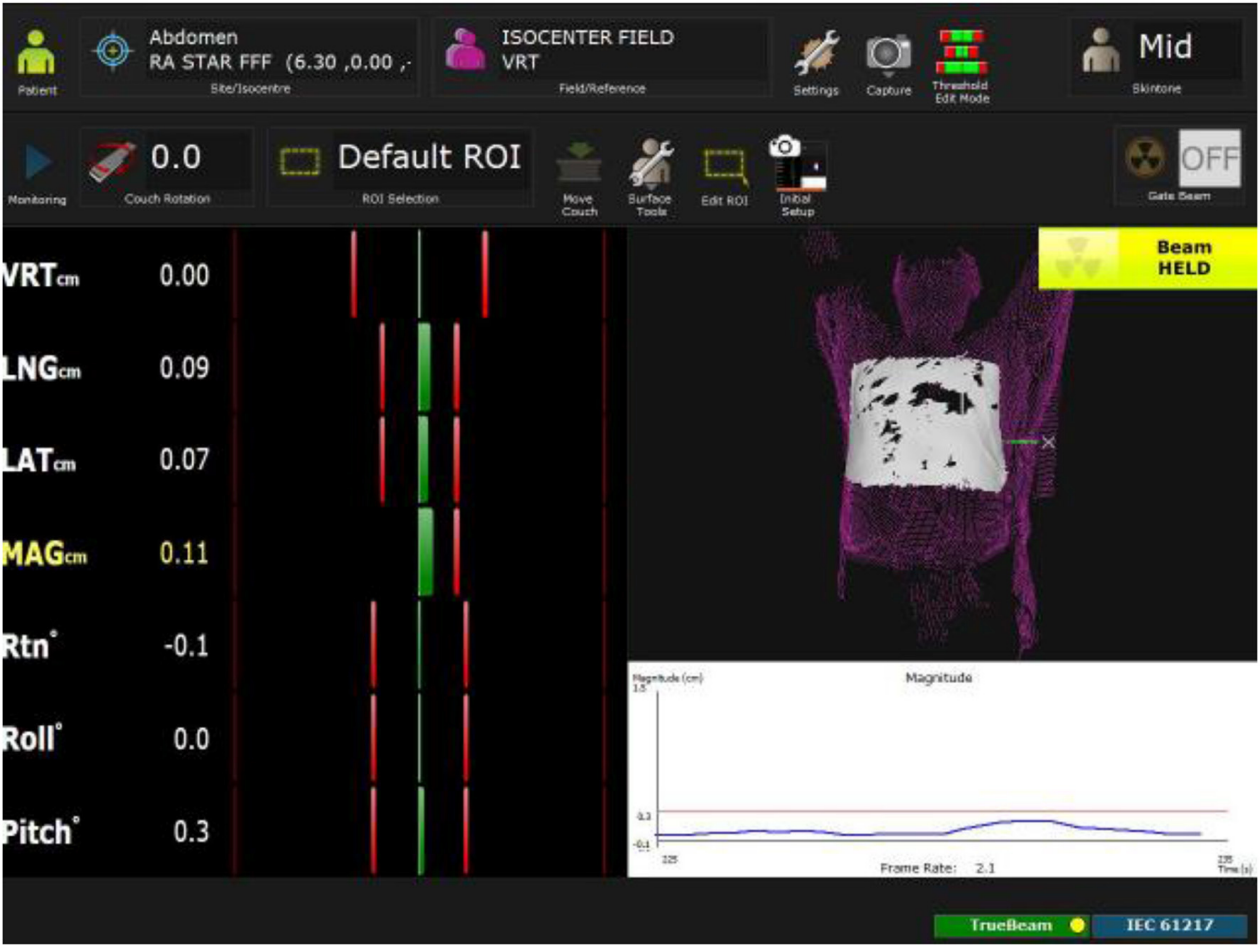
Online OSM tracking during the treatment. Tracked ROI (in withe) is defined during the reference condition process. Translational and rotational threshold are visualized as vertical red line while the actual position in highlighted as vertical green line and reported numerically in section STAR: Treatment Delivery.

OSMS was employed as surface online tracking system during the delivery. Reference image was firstly acquired, defining the tracking Region of Interest (ROI). Translational and rotational upper threshold values was set as following: vertical 0.5 cm, longitudinal 0.3 cm, lateral 0.3 cm and yaw 3°. The OSMS system was used to monitor the patient immediately prior to treatment and to track the patient during treatment in three-dimensional (3D) mode. Figure 5 shows the online OSMS tracking during the treatment and it relative setting and online recorded data.

Once OSMS was set-up, the first PA was delivered. A CBCT was acquired before each PA. OSMS recorded patient positioning and triggered the beam delivery whenever out of tolerance during the entire delivery procedure. A total dose of 25 Gy in single fraction was delivered.

Pre-treatment patient setup was performed in 15 min, including the acquisition of the OSMS reference imaging. The total duration of the whole procedure (since first CBCT to end of the last delivered MU) was 33 min and 15 s. Each CBCT acquisition took 1 min and 20 s. Patient alignment after each CBCT required <3 min, including couch correction shift application. Delivering time for each of the three PA was 3.13, 3.26, and 3.06 min, respectively.

Only one interruption occurred due to the OSMS trigger: the negative longitudinal direction exceeded of 1 mm the OSMS threshold and it caused a beam hold of <5 s. Except of this event, during the entire delivering time the maximum shifts detected by the OSMS were: vertical ± 0.1 mm, longitudinal ± 0.2 mm, lateral ± 0.1 mm, and yaw±0.4°.

No interruption due to patient related factors was necessary, in particular no ventricular arrhythmia occurred during the intervention.

Implantable Cardioverter Defibrillator (ICD) function was normal and cardiac enzymes remained stable after the radiation treatment.

**Figure 6:**
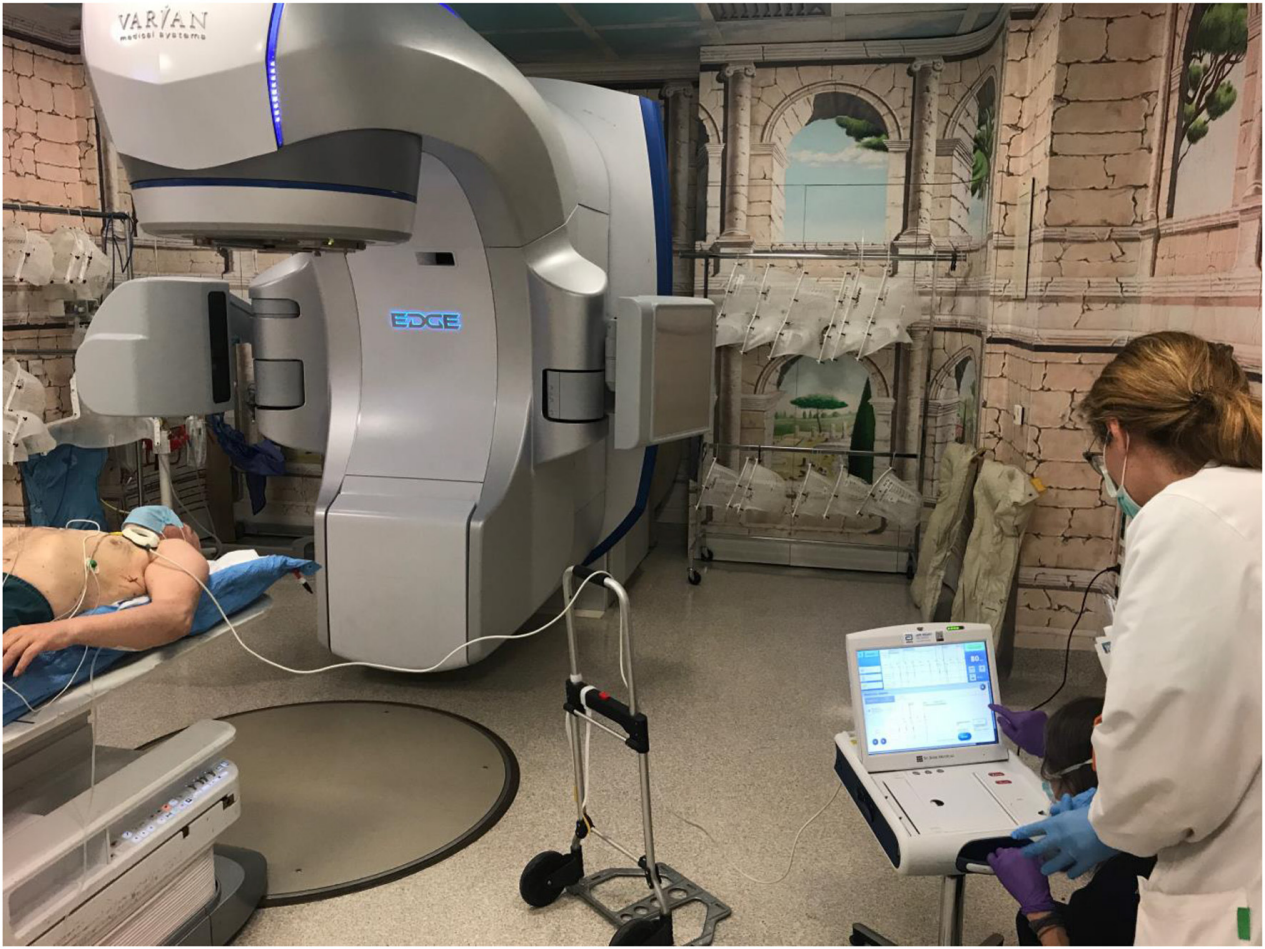
Patient monitoring within the vault before STAR delivery.

Figure 6 shows the monitoring patient preparation within the vault before STAR delivery.

## Results/Clinical Outcome

The patient was discharged from the hospital after 1 week from SBRT, without recurrence of sustained VT or VF, without ICD shocks or ATP. Before the discharge, left ventricular ejection fraction (LVEF) was 30% by transthoracic echocardiography, no pericardial effusion was detected.

The patient remained free from sustained VT or VF for the following 3 months (current follow-up by home monitoring).

## Discussion

STAR is becoming a promising, alternative, non-invasive approach for pts with VT refractory to drug and catheter ablation, unsuitable for transplantation (11). Our CR demonstrated that SBRT could be a therapeutic option in patients with ES in advance heart failure and inability to complete endo-epicardial ablation. Some reports outlined this challenging process and its clinical feasibility (2). Essentially 3 types of reports are currently available in this regard. First, preclinical reports, including both animal model evaluation of radiotherapy efficacy (12, 13) and *in silico* analysis of its treatment planning features (14). Second, clinical CRs on SBRT to manage peculiar oncological cardiac localization (mostly sarcomas) (15). Finally, some CRs (8, 9, 16), two case series (CS) (2, 7) and the result of a Phase I/II trial (6) investigated the application of SBRT in the cardiological frame (STAR). In particular, both the first report of Cuculich et al. (2) and the recent Phase I/II trial (Encore VT) from the same Group (6) suggested the efficacy and safety of such procedure. A recent systematic literature review summarized that VT was targeted with reduction in the number of episodes beyond 85% during follow-up, with an encouraging short-term safety profile (17). Nevertheless, the same analysis revealed a large heterogeneity in available study designs limiting the clinical evidence on efficacy and safety of STAR. Particularly, conflicting results have been reported by a recent CS of patients treated by GammaKnife (18), not included in the mentioned systematic review. Currently, clinical trials are ongoing (NCT04334811; CyberHeart—NCT02661048) and future evidences will provide useful insights in this regard. Personalization of such an approach could influence results. To this regard, two main issues are of high relevance: cardiac target definition by accurate mapping, and the specific details of STAR planning and delivery. The available clinical experiences adopted 3 type of devices for STAR: Cyberknife (16, 19), Image guided radiation therapy (IGRT)-Linac (2), and the MR-guided radiotherapy (MRgRT) system (9). All of them provide accurate image guidance ensuring image guided patient positioning and monitoring of both inter- and intra-fractional patients positioning errors, aiming to minimize delivery errors. Each technical approach presents peculiarities and drawbacks. Homogeneously among the published reports, the total RT prescribed dose is 25Gy in single fraction; IGRT is usually based on volumetric images. Conversely, some details about the STAR procedure are not uniformly applied, including: the margin expansion for the PTV; the reference isodose for total dose prescription; the number and sequence of the CBCT for the IGRT-Linac STAR (the most widely adopted one). Our CR focuses the issue of efficacy and practical impact of one of the highest available levels of control for inter- and intra-fractional positioning errors. Dealing with a certain wide volume, we did apply the dedicated-to-SBRT IGRT-Linac TrueBeam Edge (Varian Medical Systems, Palo Alto, CA) along with the OSMS system to continuously monitor the intra-fraction motion. The most advanced device for personalization of intra-fraction motion are CyberKnife and MRgRT. CyberKnife applies 2-dimensional IGRT during treatment; MRgRT applies volumetric IGRT, and allows an highly personalized delivery setting but its characteristics made the reported delivery time much longer that all the other ones (around 2 and half hours vs. 30–90 min) (2, 7–9). We applied continuous intra-fraction monitoring through OSMS with an acceptable total treatment delivery time of 30 min while gaining the safety of such support. In regard of inter-fractional monitoring, we repeated CBCT scan before delivering each PA of the VMAT, differently by all the available reports. The number of CBCT is not always reported in literature: some authors specified to had at least one before the beginning of the session (2, 20) or multiple ones although not for each VMAT arc (8). In our experience, combining 2 safety ensuring procedure (i.e., OSMS plus CBCT per each arc) did not resulted in much longer treatment session than literature's counterpart. Being our clinical outcome nicely similar to the literature reports (despite the PTV volume) we recommend to consider increasing as much as possible the personalization features and safety technical procedure as long as that is not significantly affecting the STAR duration. A further consideration regarding the STAR duration should also consider the Linac dose rate availability. The maximum dose rate for MRgRT does not exceeding 600 MU/min. TrueBeam Edge Linac has instead a dose rate of 1,400 MU/min for the 6 megavolt (MV) applying flattering filter free (FFF) beam, a technique significantly reducing the beam delivering time. Higher dose rate could be also delivered if 10 MV FFF beams is selected, but higher risk of cardiac device damages should be considered. However, the duration in itself shouldn't be the main parameter, but balanced into the frame of possibly obtainable outcome improvement. Future studies will clarify these issues.

## Data Availability Statement

The original contributions presented in the study are included in the article/Supplementary Material, further inquiries can be directed to the corresponding author/s.

## Ethics Statement

Written informed consent was obtained from the individual(s), and/or minor(s)' legal guardian/next of kin, for the publication of any potentially identifiable images or data included in this article.

## Author Contributions

GPi provided the concept of manuscript. MN, FCe, and LP contributed to the concept of manuscript and wrote the paper. RB, MN, FCe, LB, and LP provided the setting according to CARE Guidelines. GPe, FCr, and VV supervised the project. All Authors Contributed to this manuscript by editing, revisioning, and taking care of references.

## Conflict of Interest

The authors declare that the research was conducted in the absence of any commercial or financial relationships that could be construed as a potential conflict of interest.

## References

[1] Riley DS Barber MS Kienle GS Aronson JK von Schoen-Angerer T Tugwell P. CARE guidelines for case reports: explanation and elaboration document. J Clin Epidemiol. (2017) 89:218–35. 10.1016/j.jclinepi.2017.04.026 28529185

[2] Cuculich PS Schill MR Kashani R Mutic S Lang A Cooper D. Noninvasive cardiac radiation for ablation of ventricular tachycardia. N Engl J Med. (2017) 377:2325–36. 10.1056/NEJMoa1613773 29236642 PMC5764179

[3] Priori SG Blomstrom-Lundqvist C Mazzanti A Blom N Borggrefe M Camm J 2015 ESC Guidelines for the management of patients with ventricular arrhythmias and the prevention of sudden cardiac death: the task force for the management of patients with ventricular arrhythmias and the prevention of sudden cardiac death of the European Society of Cardiology (ESC)Endorsed by: association for European Paediatric and Congenital Cardiology (AEPC). Europace. (2015) 17:1601–87. 10.1093/eurheartj/ehv316 26318695

[4] Zipse MM Edward JA Zheng L Tzou WS Borne RT Sauer WH. Impact of epicardial adipose tissue and catheter ablation strategy on biophysical parameters and ablation lesion characteristics. J Cardiovasc Electrophysiol. (2020) 31:1114–24. 10.1111/jce.14383 32031304

[5] Santangeli P Frankel DS Tung R Vaseghi M Sauer WH Tzou WS. Early mortality after catheter ablation of ventricular tachycardia in patients with structural heart disease. J Am Coll Cardiol. (2017) 69:2105–15. 10.1016/j.jacc.2017.02.044 28449770

[6] Robinson CG Samson PP Moore KMS Hugo GD Knutson N Mutic S. Phase I/II trial of electrophysiology-guided noninvasive cardiac radioablation for ventricular tachycardia. Circulation. (2019) 139:313–21. 10.1161/CIRCULATIONAHA.118.038261 30586734 PMC6331281

[7] Lloyd MS Wight J Schneider F Hoskins M Attia T Escott C. Clinical experience of stereotactic body radiation for refractory ventricular tachycardia in advanced heart failure patients. Heart Rhythm. (2020) 17:415–22. 10.1016/j.hrthm.2019.09.028 31585181

[8] Krug D Blanck O Demming T Dottermusch M Koch K Hirt M. Stereotactic body radiotherapy for ventricular tachycardia (cardiac radiosurgery): first-in-patient treatment in Germany. Strahlenther Onkol. (2020) 196:23–30. 10.1007/s00066-019-01530-w 31673718

[9] Mayinger M Kovacs B Tanadini-Lang S Ehrbar S Wilke L Chamberlain M. First magnetic resonance imaging-guided cardiac radioablation of sustained ventricular tachycardia. Radiother Oncol. (2020). 10.1016/j.radonc.2020.01.008. [Epub ahead of print]. 32067819

[10] Benedict SH Yenice KM Followill D Galvin JM Hinson W Kavanagh B. Stereotactic body radiation therapy: the report of AAPM Task Group 101. Med Phys. (2010) 37:4078–101. 10.1118/1.3438081 20879569

[11] Zei PC Soltys S. Ablative radiotherapy as a noninvasive alternative to catheter ablation for cardiac arrhythmias. Curr Cardiol Rep. (2017) 19:79. 10.1007/s11886-017-0886-2 28752279 PMC5532420

[12] Lehmann HI Graeff C Simoniello P Constantinescu A Takami M Lugenbiel P. Feasibility study on cardiac arrhythmia ablation using high-energy heavy ion beams. Sci Rep. (2016) 6:38895. 10.1038/srep38895 27996023 PMC5171237

[13] Lehmann HI Deisher AJ Takami M Kruse JJ Song L Anderson SE. External arrhythmia ablation using photon beams: ablation of the atrioventricular junction in an intact animal model. Circ Arrhythm Electrophysiol. (2017) 10:4304. 10.1161/CIRCEP.116.004304 28408649

[14] Wang L Fahimian B Soltys SG Zei P Lo A Gardner EA. Stereotactic arrhythmia radioablation (STAR) of ventricular tachycardia: a treatment planning study. Cureus. (2016) 8:e694. 10.7759/cureus.694 27570715 PMC4996541

[15] Gabani P Fischer-Valuck BW Robinson CG Wilson DB Michalski JM. Stereotactic body radiation therapy for the treatment of primary cardiac angiosarcoma causing hemodynamic instability. Pract Radiat Oncol. (2019) 9:5–8. 10.1016/j.prro.2018.07.004 30611463

[16] Loo BWJr Soltys SG Wang L Lo A Fahimian BP Iagaru A. Stereotactic ablative radiotherapy for the treatment of refractory cardiac ventricular arrhythmia. Circ Arrhythm Electrophysiol. (2015) 8:748–50. 10.1161/CIRCEP.115.002765 26082532

[17] van der Ree MH Blanck O Limpens J Lee CH Balgobind BV Dieleman EMT. Cardiac radioablation-A systematic review. Heart Rhythm. (2020) 17:1381–92. 10.1016/j.hrthm.2020.03.013 32205299

[18] Gianni C Rivera D Burkhardt JD Pollard B Gardner E Maguire P Stereotactic arrhythmia radioablation for refractory scar-related ventricular tachycardia. Heart Rhythm. (2020) 17:1241–8. 10.1016/j.hrthm.2020.02.036 32151737

[19] Zeng LJ Huang LH Tan H Zhang HC Mei J Shi HF. Stereotactic body radiation therapy for refractory ventricular tachycardia secondary to cardiac lipoma: a case report. Pacing Clin Electrophysiol. (2019) 42:1276–9. 10.1111/pace.13731 31116434

[20] Bhaskaran A Downar E Chauhan VS Lindsay P Nair K Ha A. Electroanatomical mapping-guided stereotactic radiotherapy for right ventricular tachycardia storm. HeartRhythm Case Rep. (2019) 5:590–2. 10.1016/j.hrcr.2019.09.007 31890583 PMC6926178

